
JOURNAL INFORMATION
==============================
NLM Title Abbreviation: Behav Neurol
Journal ID: Behav Neurol
NLM Journal ID: 8914585
Journal ID: BN

Behavioural Neurology

ISSN: 0953-4180
EISSN: 1875-8584
Publisher: Wiley

ARTICLE INFORMATION
==============================
PMCID: PMC5377964
PMID: 21447891
DOI: 10.3233/BEN-2011-0323
Article ID: 646483
Article version: 1
Subjects: Editorial

Epilepsy and Disorders of Consciousness

Cavanna Andrea Eugenio 1 2 *
1 Department of Neuropsychiatry University of Birmingham and BSMHFT Birmingham UK
2 Department of Neuropsychiatry Institute of Neurology and UCL London UK
*Andrea Eugenio Cavanna: a.cavanna@ion.ucl.ac.uk
Print publication date: 2011
Electronic publication date: 2011 Mar 29
Volume: 24
Issue: 1
First page: 1
Last page: 1
Received 2011 Mar 23; Accepted 2011 Mar 23
Copyright: Copyright © 2011 Hindawi Publishing Corporation and the authors.
Copyright year: 2011
License: This is an open access article distributed under the Creative Commons Attribution License, which permits unrestricted use, distribution, and reproduction in any medium, provided the original work is properly cited.
License URL: https://creativecommons.org/licenses/by/3.0/

==============================
Behavioural Neurology 24 (2011) 1
DOI 10.3233/BEN-2011-0323
IOS Press

Editorial

### Epilepsy and Disorders of Consciousness

Andrea Eugenio Cavanna[a,b,*]
[a]*Department of Neuropsychiatry, University of Birmingham and BSMHFT, Birmingham, UK*
[b]*Department of Neuropsychiatry, Institute of Neurology and UCL, London, UK*

This issue of Behavioural Neurology is devoted to the investigation of the long recognised link between epilepsy and disturbances of consciousness. Interest in this topic has witnessed an unprecedented scientific renaissance over the last few years, for two orders of reasons. First, most types of epilepsy are characterised by specific alterations in the level of general awareness and/or the subjective contents of consciousness, which are central aspects of patients' ictal experiences and can now be assessed and quantified with newly developed psychometric tools. Second, the investigation of what Hughlings-Jackson called the 'occasional, sudden, rapid and local discharges of grey matter' has started to shed light on the brain mechanisms underlying pathophysiological states of altered consciousness, thanks to the development of sophisticated neurophysiological and neuroimaging techniques. The implementation of combined electroencephalography and functional magnetic resonance imaging (EEG-fMRI) is just an example of this ongoing revolution. Therefore, the fact that this special issue is appearing in Behavioural Neurology reflects the surge of interest from both neurologists and neuroscientists. In this issue, epilepsy experts from around the world have contributed their insights by reviewing the latest developments in clinical and experimental research on epilepsy and consciousness.

The first set of contributions focuses on the phenomenological analysis of ictal alterations of consciousness in patients with epilepsy (Johanson), which is accompanied by specific behavioural (Mula) and cognitive (Krishnamoorthy) changes. The second group of papers presents our current knowledge of the brain mechanisms underlying altered consciousness in both focal (Bagshaw) and generalized (Seri) seizures. The sudden alterations of consciousness caused by absence seizures are of particular conceptual relevance, and are also addressed from a theoretical perspective (Bayne). The following section deals with two emerging scientific paradigms which hold promise in refining our understanding of the neural bases of epilepsy-induced alterations of consciousness. These are the definition of the 'default mode network' (Blumenfeld), which has been shown to have a central role in neuropsychiatric conditions characterised by alterations of consciousness, and the conceptual framework of the 'global workspace theory of consciousness' (Bartolomei), which provides a useful model for understanding both normal and altered conscious states. Finally, the papers by Bagary, Selai and Reuber address specific issues related to epilepsy and consciousness in clinical practice, namely the recent applications of neurostimulation techniques, the impact of seizure-induced alterations of consciousness on the quality of life of patients, and consciousness in non-epileptic attack disorder.

The quality of this collective effort confirms that the study of epilepsy-induced alterations of consciousness is central to the discipline of behavioural neurology. This was already recognised over a century ago by the fathers of modern behavioural neurology. To quote Hughlings-Jackson again, his 1888 article "*On a particular variety of epilepsy* (*'intellectual aura'*)*, one case with symptoms of organic brain disease*" proudly stated that "*He who is faithfully analysing many different cases of epilepsy is doing far more than studying epilepsy. The highest centres* (*'organ of mind'*)*, those concerned in such fits, represents all, literally all, parts of the body sensorially and motorially, in most complex ways, in most intricate combinations, etc. A careful study of many varieties of epileptic fits is one way of analysing this kind of representation by the 'organ of mind'*." We believe that this journal's special issue on epilepsy and consciousness has proved him true.

*Address for correspondence. E-mail: a.cavanna@ion.ucl.ac.uk.

ISSN 0953-4180/11/$27.50 © 2011 – IOS Press and the authors. All rights reserved
